# Perceptual-motor styles

**DOI:** 10.1007/s00221-021-06049-0

**Published:** 2021-03-06

**Authors:** Pierre-Paul Vidal, Francesco Lacquaniti

**Affiliations:** 1 CNRS, SSA, ENS Paris Saclay, Université de Paris, Centre Borelli, 75005 Paris, France; 2 Institute of Information and Control, Hangzhou Dianzi University, Hangzhou, China; 3 Department of Systems Medicine, Center of Space Biomedicine, University of Rome Tor Vergata, 00133 Rome, Italy; 4 Laboratory of Neuromotor Physiology, Santa Lucia Foundation IRCCS, 00179 Rome, Italy

**Keywords:** Redundancy, Adaptation, Plasticity, Variability

## Abstract

Even for a stereotyped task, sensorimotor behavior is generally variable due to noise, redundancy, adaptability, learning or plasticity. The sources and significance of different kinds of behavioral variability have attracted considerable attention in recent years. However, the idea that part of this variability depends on unique individual strategies has been explored to a lesser extent. In particular, the notion of style recurs infrequently in the literature on sensorimotor behavior. In general use, style refers to a distinctive manner or custom of behaving oneself or of doing something, especially one that is typical of a person, group of people, place, context, or period. The application of the term to the domain of perceptual and motor phenomenology opens new perspectives on the nature of behavioral variability, perspectives that are complementary to those typically considered in the studies of sensorimotor variability. In particular, the concept of style may help toward the development of personalised physiology and medicine by providing markers of individual behaviour and response to different stimuli or treatments. Here, we cover some potential applications of the concept of perceptual-motor style to different areas of neuroscience, both in the healthy and the diseased. We prefer to be as general as possible in the types of applications we consider, even at the expense of running the risk of encompassing loosely related studies, given the relative novelty of the introduction of the term perceptual-motor style in neurosciences.

## Introduction

Goal-directed movements, such as reaching, throwing, postural responses or locomotion, involve complex sensorimotor transformations that require the integration of multiple sensory inputs and the coordination of multiple motor outputs (Soechting and Flanders 1992; Massion 1994; Lacquaniti 1997; Ting and McKay 2007; Peterka 2002; Guerraz and Bronstein 2008; Carver et al. 2006). Given the complexity of these processes, it is not surprising that they almost never yield stereotypical responses, being instead associated with multiple solutions across repetitions and individuals even under identical initial conditions. Before considering the issues of variability and style, a few preliminary, relevant points must be considered.

First, there is the issue of redundancy. The number of degrees of freedom (DOFs) of our musculoskeletal system is very large. By assuming that there are 148 movable bones connected by joints in the human skeletal system and taking into account the kinematic constraints, the total estimated number of DOFs corresponds to 244 (Prilutsky and Zatsiorsky 2002). This number greatly exceeds the 6 DOFs required to place a body segment in a desired position with a desired orientation, for example when placing the hand over a computer mouse or the foot over a staircase step. Thus, given the redundant DOFs, there is an infinite number of different kinematic configurations of the body compatible with a given motor task. Moreover, since there are about 630 skeletal muscles in the human body, an average of 2.6 muscles acts upon each kinematic DOF. Given that at least two muscles are available at each articular DOF, there is an infinite number of muscle force combinations that can produce a required joint torque (Prilutsky and Zatsiorsky 2002). The high redundancy of the musculo-skeletal system, coupled with the morpho-functional diversity of people (body height, mass, shape), makes it highly unlikely that any two different persons will adopt the same postural configuration for the same task under identical circumstances.

Secondly, behavior shows a great deal of adaptability driven by evolution. Our sensorimotor control cannot rely solely on an invariant repertoire of muscle responses. Stereotyped responses were not even sufficient when our distant ancestors lived in the aquatic environment some 400 million years ago.

Another relevant point concerns the plasticity of perceptual-motor responses following training or pathologies. Highly intensive training can profoundly alter our sensorimotor transformations to improve our performance in sports, for example. However, this does not guarantee that these changes are globally optimal in the long term, as the musculoskeletal injuries of joggers all too often demonstrate. In the same way, the occurrence of pathologies can profoundly alter motor responses and the underlying sensorimotor transformations. Therefore, individual longitudinal monitoring or follow-up of persons using quantitative approaches based on individual markers of behavior becomes imperative (Vidal et al. 2020).

Genetic differences between individuals, developmental stage, and age are other important factors that contribute to variability.

## Different kinds of behavioral variation

Given the above premises, it is not surprising that sensorimotor behavior is typically characterized by a variety of implementation and expression solutions. Behavioral variety can occur along a continuum or it can involve discrete categories. Although the border between these two forms of variation is not always sharp, discrete categories are identifiable when measurable parameters allow clustering individuals or behaviors in different groups (e.g., Schorer et al. 2007; Maselli et al. 2019). Clustering requires that the individuals or behaviors belonging to the same group have parameter values more similar to each other than to those in the other groups. There is a wealth of statistical techniques for optimal clustering, such as those based on the comparison of within-cluster distances with between-clusters distances (Gan et al. 2007). In addition, a variety of similarity measures are available to classify behavior and individuals (e.g. nearest-neighbor statistics, Van Der Maaten 2014), especially when the existence of clusters can be an unwarranted approximation.

## Variability

Variability is ubiquitous but it takes different forms with different neural origin and different functional significance. Some of this variability is due to noise in neural spike trains, but some variability is principled. Variability in sensory estimation can be propagated through sensorimotor circuits, ultimately causing motor variability (Lisberger and Medina 2015). Each repetition of a motor action corresponds to a potentially different neural state, defined probabilistically within high-dimensional distributed networks (Shenoy et al. 2013).

Intra-individual, inter-trial variability due to various sources of noise (at the level of planning, execution or sensory feedback) is a fundamental characteristic of biological behavior and of the underling neural activity (Faisal et al. 2008). Even professional athletes -such as Major League baseball pitchers- exhibit trial-to-trial variability in their performance (Chaisanguanthum et al. 2014), although they tend to have more stable movement patterns than novices (Müller and Sternad 2004; Newell et al. 2006). The motor variability that interferes with performance is undesirable, and the central nervous system (CNS) may try to compensate for it by means of optimal control (Harris and Wolpert 1998; Todorov and Jordan 2002). The variability that does not interfere with performance but contributes to redundant control is not compensated (Lacquaniti and Maioli 1994; Scholz and Schoner 1999). One should also consider that behaviour can be learnt from two separate systems: one system creates habitual patterns based on past successful associations of actions with stimuli and context, and another system selects actions to best achieve a goal given the current stimuli and context (Robbins and Costa 2017). Practice promotes habit formation, and at the same, it modulates the likelihood of habit expression (Hardwick et al. 2019).

Variability can be more than just noise when people learn a new task and take advantage of inter-trial variability to explore the solution space by means of reinforcement strategies (Chaisanguanthum et al. 2014; Dhawale et al. 2017). Importantly, different subjects may show different levels of inter-trial variability during learning, which are consistent across movements and effectors, indicating the existence of individual traits. Thus, subjects with higher initial levels of task-relevant inter-trial variability tend to learn reaching tasks faster than subjects with lower inter-trial variability (Wu et al. 2014).

Individual hallmarks of this kind may underlie excellence in highly specialized skills such as those involved in sports or artistic performances (Yarrow et al. 2009). Age is also an important factor contributing to inter-individual variability. Thus, a recent study showed that elderly and young individuals rely on different aspects of motor variability to drive learning (Cheung et al. 2020). In the latter study, the score in a virtual bowling task correlated with the changes of timing variability of muscle activation in elderlies, while the score correlated with the variability changes of synergy magnitude in young adults. Notice further that, when the mode of rehabilitation training allows variability of limb trajectory, recovery from a lesion of the spinal cord is improved relative to training with a fixed trajectory (Ziegler et al. 2010).

## Style

The kind of variability that tends to be associated with different individuals comes close to overlap with individual style. However, the variability of performance may be a transient feature of a specific behavior, while *style* refers to a relatively stable, consolidated feature of a behavior associated with a given context and developmental stage. Since most studies of sensorimotor variability describe features of behavior without considering whether these features are ephemeral or lasting, they may miss the identification of perceptual-motor styles in the sense we discuss in this review. However, it is quite likely that a number of instances of variability represent stable individual traits (e.g. Haar et al. 2017), and thus may be considered analogous to individual styles.

**Fig. 1:**
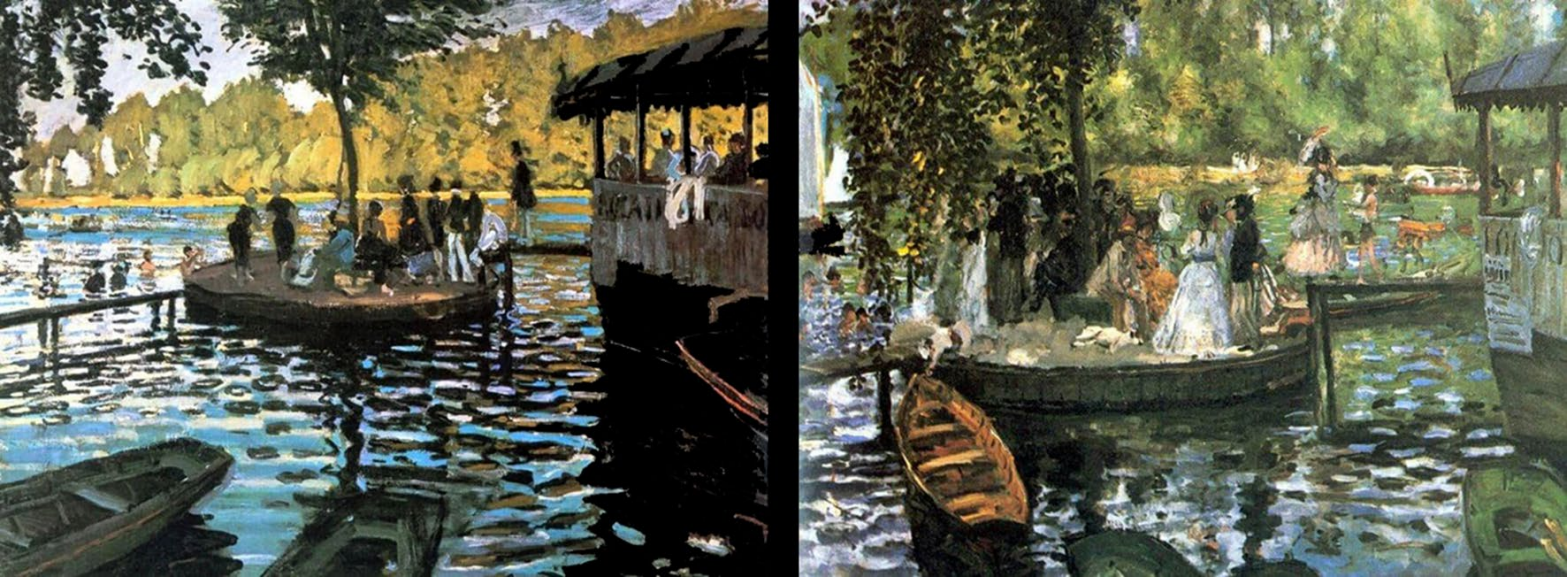
La Grenouillère: same subject, two painters, two styles. The Frog Pond and Island were painted around the same time (1869) by the two friends, Claude Monet (left) and Pierre-Auguste Renoir (right)

While the keyword of variability recurs quite frequently in the literature on sensorimotor behavior, the keyword of style is much less common in this realm. In fact, the notion of style is traditionally employed in the field of arts and entertainment to indicate the “how” a piece of work is realized, rather than the “what”, “why”, “when” or “where” (McMahon 2003). For instance, according to Fernie (1995), style is a "distinctive manner which permits the grouping of [artistic] works into related categories”. Interestingly, the term style stems etymologically from stylus, the Latin word for an ancient writing utensil. With time, the metonymical usage of the term included the rhythm of handwriting independent of the written content and subsequently encompassed the artistic style in the general sense used today (Pinotti 2012). Therefore, style is historically rooted in sensorimotor control. Indeed, according to the art historian Ernst Gombrich (1998), “style is any distinctive, and therefore recognizable, way in which an act is performed or an artefact made or ought to be performed and made”. Nevertheless, even in art history, the definition of style is not univocal. According to McMahon (2003), the term can be used to denote alternatively: (i) a period in history like the Early or High Renaissance; (ii) an artistic movement like Mannerism or Futurism; (iii) various developmental stages in an artist's oeuvre; (iv) the artist’s point of view which may be ascertained from other than discernible properties in the artwork; and (v) a set of formal characteristics which cuts across periods, movements and individual oeuvres. The formal characteristics differentiate one artist’s style from another one within a more general class, such as the impressionism (Fig. 1).

Operational definitions of *style* are still more difficult in physiology because its scientific inquiry requires objective, quantitative assessments. In principle, quantitative discrimination of styles depends on a wide (potentially unlimited) set of different parameters. In the following, we will consider different ways to identify perceptual-motor styles. It should be stressed that, just as in the case of arts and entertainment, also in physiology a given style is not necessarily unique to an individual, but it can be associated with different people according to the specific context, for instance when performing a given task or expressing a specific emotion. Only when a style is unique to an individual does it become a reliable biometric identifier, that is, a measurable behavioral feature that can be used to label individuals. Notice, however, that in forensic science the concept of individuality is prudently taken in relative, not absolute sense since it is impossible to prove that a given human characteristic is unique to a given person without checking every other person in the world (Saks and Koehler 2008). The goal, then, is that of establishing individualization without claiming universal uniqueness (Kaye 2009). Furthermore, just as a given style can be associated with different persons, a given person may adopt different styles depending on the context or developmental stage.

In the following, we will retain the definition of perceptual-motor style as any distinctive and recognizable way in which an action is performed or perception is processed. This perceptual-motor style may be typical of a person, group of people, context, task or age. It may be determined genetically or as a result of development, learning, pathology. To be more comprehensive, we shall review both studies in which the term style was employed by the authors as well as studies in which the term of variability was used but, in fact, it referred to a phenomenology that would fall under the current definition of style. We do not claim to be exhaustive, since the pertinent literature would be potentially enormous, and we apologize for the inevitable omissions. At the end, we will consider some potential neural underpinnings of perceptual-motor styles.

## The sensory side

### In healthy persons

Witkin and collaborators were among the first scientists to use the term *style* in a perceptual-cognitive context (for a thorough review of cognitive styles in the context of psychology including an historical account, see Kozhevnikov 2007). To assess the subjective upright in space, Witkin and Asch (1948) introduced the Rod and Frame Test in which a rod and an external wireframe are rotated independently by variable angles. Based on the response to the test, subjects were classified into two distinct categories, each denoted as a specific cognitive style. Subjects who aligned the rod so that it leaned in the direction of the tilted frame were defined as field-dependent, since they relied on the visual field defined by the frame to judge the vertical. Instead, subjects who were able to align the rod close to the vertical independently of the wireframe orientation were defined as field-independent. To judge the vertical, the latter group of subjects relied more on vestibular and postural cues about the direction of the pull of gravity on the body. Since its introduction, the Rod and Frame Test has been used to assess perceptual-cognitive styles in several different conditions, from educational to sports and clinical contexts (e.g. Chan and Yan 2018; Evans et al. 2013).

Moreover, the pioneering results obtained by Witkin and collaborators with this text proved critical for the first elaboration of multisensory integration for the perception of the upright by Gibson (1952), who suggested that the visual vertical can be determined by a weighted combination of visual and postural cues. He argued that, in case of a discrepancy, the brain learns to use the reliable cues and to neglect the unreliable ones.

In fact, the existence of a sensory side of perceptual-motor style can be accounted for by the fact that several sensory systems are involved in generating an internal representation of the body in space and the perception of its own movement (Merfeld et al. 1999; Green and Angelaki 2010; Lacquaniti et al. 2014). Visual information determines the orientation of objects in space and the detection of body movements, including postural oscillations at rest (Lishman and Lee 1973; Prioli et al. 2005). Somatosensory information generated by muscle, joint and skin receptors encodes data on the relative position of the head, trunk and limbs in space (Barela et al. 2009; Allison et al. 2006; Jeka et al. 2000). Finally, vestibular information encodes the position as well as linear and angular accelerations of the head, thus helping to inform the brain of its orientation and movements in relation to space (Peterka and Benolken 1995). Continuous reweighting of these three types of sensory information is necessary for effective, flexible, and context-sensitive postural control, as shown in numerous studies (Mahboobin et al. 2005; Angelaki and Cullen 2008; Angelaki et al. 2009; Palluel et al. 2011; Block and Bastian 2011; Goodworth and Peterka 2012; Hwang et al. 2014; Assländer and Peterka 2014; Assländer 2016; Logan et al. 2014; Cyr et al. 2019; Dakin et al. 2020) conducted since the pioneering publication of Nashner (1976). The multi-sensory integrations that underlie our perception of our environment and our motor control are not simple algebraic additions of the sensory information available. Instead, they are based on a process of combining sensory inputs where the weight of each type of information is proportional to its relative reliability in a given context (Kabbaligere et al. 2017). For example, if a person uses cutaneous information generated by the sliding of the hand in contact with a fixed surface to learn about her/his body movement, it is this haptic information that will determine the assessment of her/his own movement as a priority (Harris et al. 2017). This explains why even minimal tactile cues are so effective in maintaining postural stability (Oie et al. 2002; Honeine 2015).

However, sensorimotor transformations and their weightings in contexts of sensory conflict or simply in unusual contexts can be difficult to interpret. Static equilibrium is usually quantified by oscillations of the center of pressure (COP) within the base of support. Increased variability of the COP, as well as an increase in its excursion and velocity are often considered an alteration of postural control. Nevertheless, these same COP oscillations may reflect an exploratory mechanism, necessary to provide increased feedback to the CNS (Schieppati et al. 2002). Methods used for analyzing random-walk-like stochastic patterns have been applied to COP trajectories to understand individual differences in quiet stance (Maurer and Peterka 2005). It should also be noted that the individual characteristics of static posture at rest do not allow us to prejudge the course of compensatory postural adjustments caused by a postural perturbation (Moya et al. 2009; Sell 2012). In this vein, the results of studies performed on postural control in space indicate a strong heterogeneity among astronauts in the adaptation of their perceptual-motor style to microgravity: their sensitivity on the plantar sole increases, which seems logical since vestibular information is profoundly modified, but static postural control is not correlated with this increase (Strzalkowski 2015). Similarly, contrary to what might be expected, vection sensitivity and latency are not uniformly modified in astronauts (Mueller et al. 1994; Oman et al. 2003).

### In disease

Again, the interpretation of increased COP movement in pathology may be indicative of either a deficit in static postural control or an attempt to increase sensory feedback, or both. The same problem therefore arises, but with greater acuity since the clinician will adopt radically different rehabilitation strategies depending on the interpretation (Geurts et al. 2005).

### Vestibular pathologies

The abundance of literature on the subject of vestibular compensation does not allow an exhaustive discussion of the subject in this article. Numerous reviews have been written on the subject (Thigilet et al. 2019; Lacour et al 2016) and their rehabilitation (Sulway and Whitney 2019; Sienko et al. 2018) to cite the most recent. The reader is referred to these for a more in-depth study of the topic. We will limit ourselves here to addressing the problem of weighting sensory inputs during a vestibular deficit. As early as 1982, Nashner et al. pointed out that the main problem for patients with vestibular deficits was their inability to weigh sensory information. In other words, these patients would be handicapped not so much by the loss of vestibular information as by their inappropriate responses to proprioceptive and visual information. Nashner concluded that vestibular information provides a necessary internal frame of reference for the interpretation of visual and proprioceptive afferents, a conclusion supported by a study by Creath et al. (2002).

It is also well established that vestibular deficits can lead to increased sensitivity to visual (Cheung et al. 1989) and proprioceptive stimulation of the lower limbs (Faralli et al. 2009). An interesting question is the dynamics of this visual prevalence as a function of the dynamics of loss of vestibular afferents. An abrupt loss of vestibular afferents would be less likely to cause a strong visual dependence than a progressive loss. Conversely, postural deficits would be more accentuated (Tjernström et al. 2018). The nature of the vestibular lesion also has an influence on the vestibular syndrome (Magnusson and Padoan 1991) as well as the activity of the person (Parietti-Winkler et al. 2016).

### Pathologies of the somatosensory system

Proprioceptive afferences from the plantar side of the feet naturally play an important role in postural and locomotor control. A study by Pasma et al. (2012) shows that proprioceptive information from each leg is independently weighted according to its reliability. The contribution of proprioceptive information is clinically assessed by testing static postural control on foam mats (Schut et al. 2017). When proprioceptive afferents are impaired, is the vestibular information sufficient to control static posture? The answer is yes: in a subject with sensory polyneuropathy that resulted in a significant loss of positional awareness of her whole-body, Blouin et al. (2007) observed that sitting posture without back or arm support was maintained when the eyes were closed and both legs were dangling. Subjects with peripheral neuropathies respond much more strongly to galvanic vestibular stimulation than healthy subjects (Day and Cole 2002; Horak and Hlavacka 2001). Also, during peripheral neuropathies, compensation strategies differ considerably from one subject to another (Bunday and Bronstein 2009). Finally, it is relevant that adolescents with idiopathic scoliosis have difficulties in weighting sensory information (Simoneau et al. 2006), as is the case with vestibular patients (Nashner et al. 1982).

### Stroke

**Fig. 2:**
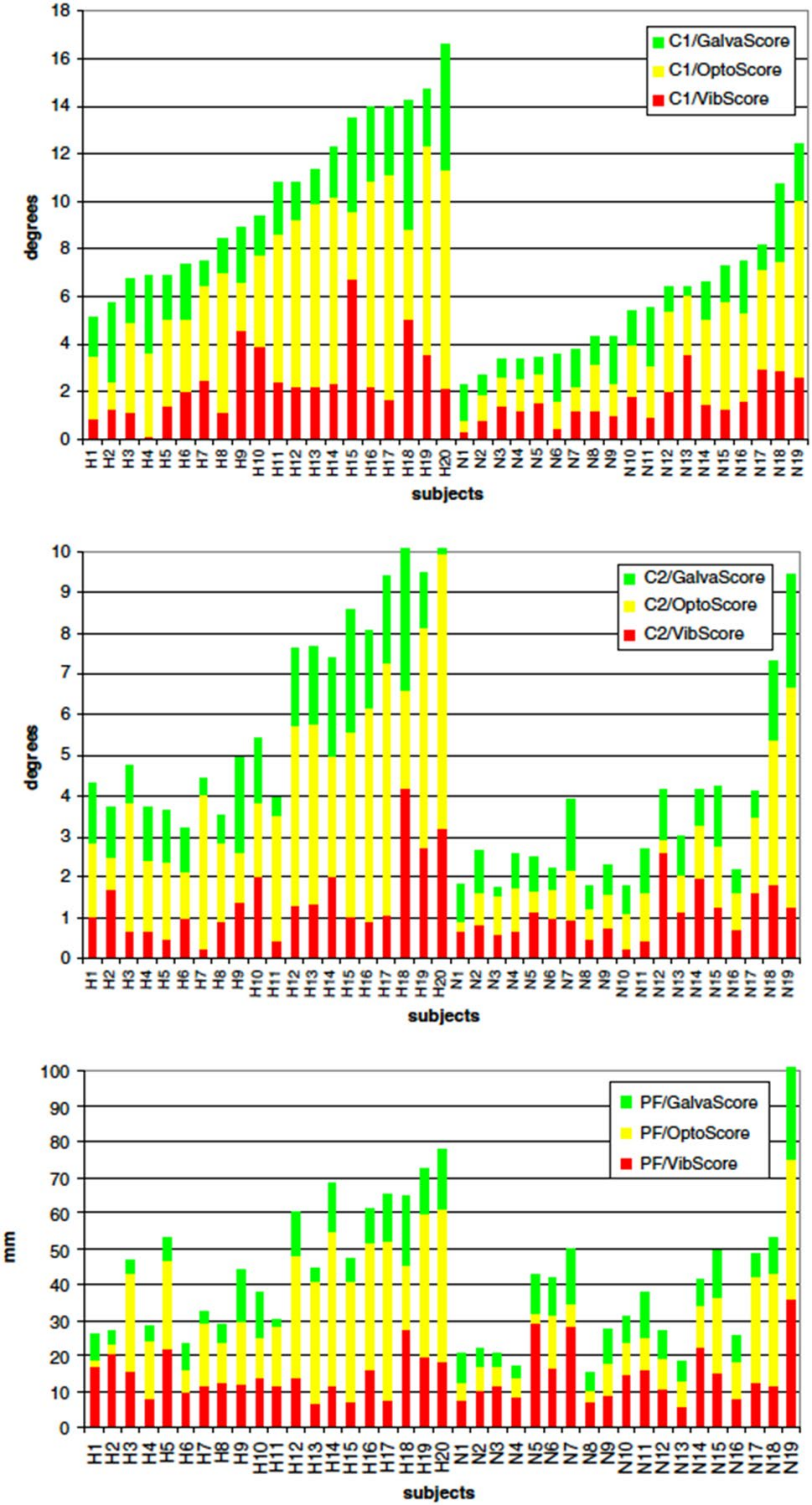
Interindividual variability of the responses to sensory stimulations in control and stroke patients. Composite scores (in degree or mm) of different subjects during optokinetic (red), vibratory (blue) and galvanic (green) stimulations recorded by the inertial sensor placed on the head (top panel, C1), trunk (middle panel, C2) and the platform (bottom panel, PF). The 20 hemiparetic subjects are labeled as H1–H20 and the control subjects as N1–N20). Modified with permission from Bonan et al. (2013)

Stroke patients are highly visually dependent (Corriveau et al. 2004; Bonan et al. 2004, 2006, 2013, 2015; Yelnik et al. 2006; Tasseel-Ponche et al. 2017). This visual dependence is also accompanied by an increased sensitivity to proprioceptive and vestibular information (Marsden et al. 2005). Studies on the weighting of sensory input following stroke also demonstrate significant inter-individual variability between patients (Bonan et al. 2013, 2015). Some subjects are insensitive to sensory stimuli, while others have static postural control that is highly impacted by one, two, or three types of stimuli. Between these two groups, many patients are moderate responders (see Fig. 2).

## Conclusion

Postural control is based on the generation of joint torques involving feedback loops (Alberts et al. 2016; Ravaioli et al. 2005; Peterka 2002; Peterka and Loughlin 2004; Kluzik et al. 2007; Cenciarini and Peterka 2006; Carver et al. 2005, 2006). A wide inter-individual variety of sensorimotor transformations seems to be the rule in both normal or pathological subjects, whether they involve postural responses to trunk acceleration (Vibert et al. 2001), to optokinetic stimulation (Sasaki et al. 2002), to eye closure (Lacour et al. 2016), or during the rod and frame test (Isableu et al. 2003). The instructions given to subjects also influence sensorimotor transformations (Fitzpatrick et al. 1992).

In congenital or acquired pathologies, structural and functional changes of the CNS are the rule. Whether this post-lesional plasticity is beneficial remains to be demonstrated. Indeed, it often appears pejorative for the functional prognosis, at least in the case of postural control. For example, although people suffering from congenital blindness present significant structural changes at the cortical level, their postural control remains deficient (Parreira et al. 2017 for a review on the subject).

This highlights the important role of training (Herssen and Mc Crun 2019) and rehabilitation in the case of sensory deficits to improve posture and locomotion control. Several studies suggest that a rehabilitation program based on visual deprivation could promote the use of somatosensory and vestibular afferents and thus reduce visual dependence (de Haart et al. 2004; Bonan et al. 2004; Di Fabio and Badke 1991). In addition, in the aftermath of a stroke, sensory stimulation can be used to normalize postural control and reduce the extent of postural deficits, including visual stimulation (Tilikete et al. 2001; Rode et al. 2006; Bonan et al. 2016), vestibular stimulation (Rode et al. 1997, 2005), and somatosensory stimulation (Pérennou et al. 1999, 2001; Pérennou 2006).

Notice that the concept of perceptual-motor styles has also proven useful in the analysis of performance in children with minimal brain dysfunction (Cakirpaloglu and Radil 1992). Thus, in a video-game, small and fast targets were often missed by brain-damaged children but not by healthy children. Also in this case, rehabilitation would benefit from visuomotor training protocols. In this respect, it has been shown that different stroke patients have different attention strategies during motor imagery rehabilitation (Sakurada et al. 2017). The ability to identify these individual strategies can therefore be useful in rehabilitation.

### The motor side

Different kinds of movement features specific to an individual have been described from kinematic or kinetic data. Thus, it has long been known that the idiosyncratic shape of the signature of each person tends to be preserved through wide changes in execution size, speed and even effector (right or left hand, foot, mouth) and it is easily recognizable whether it is written with a pen on paper, with a stylus on a tablet, or a brush on a billboard (so-called motor equivalence). Since handwriting is highly individual, it can be used as a reliable biomarker (Plamondon and Srihari 2000). For instance, individual discriminability was determined at 98% confidence using macro-features of the handwritten text of 1500 subjects (Srihari et al. 2002).

In the following, we will use human gait as the main paradigm to illustrate critical movement features because much data exist for this behavior, but we will consider other examples of movements as well.

### Visual recognition of individuals

As remarked at the outset of this article, individuals exhibit appreciable, often idiosyncratic variations in performing motor actions. These idiosyncrasies can be picked up perceptually even from limited visual cues. Thus, a common feeling is that we can recognize a familiar person from afar by looking at the way this person walks. It has been demonstrated objectively that recognition still occurs with very impoverished cues, in so far as viewers can recognize themselves and their friends from walking movements (Cutting and Kozlowski 1977) or arm movements (Hill and Pollick 2000) displayed as time sequences of point-lights corresponding to the main body joints (Johansson 1975). Recognition becomes chance-level when these animations are displayed in unusual orientations, such as upside-down (Loula et al. 2005). These abstract displays are devoid of familiarity cues, size and shape cues, or other non-kinematic sources of information. Biological motion stimuli such as those of point-light displays are interpreted by the brain based on local motion cues from the limbs, as well as on changing body configuration (Blake and Shiffrar 2007). These configural and motion cues are processed in dedicated brain regions, processed respectively in ventral and dorsal cortical pathways (Giese and Poggio 2003; Jastorff and Orban 2009; Maffei et al. 2015). A specific mechanism for action recognition has been suggested based on the discovery of mirror neurons in the ventral premotor cortex and a network of interconnected brain regions (Rizzolatti and Craighero 2004). The mechanism consists in the fact that, when we watch someone performing an action, our brain simulates the performance of the observed action (motor simulation theory). The mechanism hinges on the specific properties of visually responsive motor neurons, called mirror neurons. Thus, premotor cortex, parietal and occipito-temporal regions are activated in functional imaging studies when expert dancers view movements that they have been trained to perform (Calvo-Merino et al. 2005), or when naïve observers view silent video-clips of speech recorded in their familiar language as opposed to a non-familiar language (Maffei et al. 2020).

However, the features of visual motion that are used for individual human recognition are still incompletely understood. Pollick and Paterson (2008) remarked that style recognition requires first categorizing the movement type (walking, dancing, drinking, lifting, etc.), and then recognizing gender, ethnicity, age, emotion, identity. There is no single source of information about individual movement style, but a rich potential feature space available for recognition. Notice, however, that the perception of human identity and style is not always accurate since, in fact, it is often only slightly above chance-level (Cutting and Kozlowski 1977; Loula et al. 2005). However, this lack of accuracy does not necessarily imply that the information is not present in the animation per se. Unsurprisingly, recognition is much better when human movements are shown in full under natural viewing conditions, rather than as abstract point-light displays (O'Toole et al. 2011).

### Automatic video recognition of individuals

The recent rapid developments of various techniques to monitor human movements on-line and cheaply have led to the proposal to use individual gait recognition as a biometric trait in several applicative fields beyond biomedicine (e.g., Boyd and Little 2005; Han and Bhanu 2005; Sprager and Juric 2015). The use of gait for human identification is still very recent as compared to methods based on fingerprints, voice, or face recognition. However, in contrast with other biometric variables, gait has the advantage of being difficult to imitate or camouflage. Moreover, it can be monitored remotely without the need for cooperation, contact or high image resolution. On the other hand, gait identification is made difficult by the presence of several confounding factors, such as variations due to walking speed, footwear, terrain, fatigue, injury, or passage of time. In addition, the caveat about the uniqueness of biometric parameters mentioned above naturally applies to gait parameters.

In automated surveillance and security scenarios, the ideal goal, not yet reached by current methods, would be to analyze the collected video data by means of machine-learning algorithms, detect abnormal behavior, determine the identities of all persons in the scene, track the suspects, and warn before an adverse event happens (Zhang et al. 2011). Current gait recognition techniques rely on the analysis of spatial and/or temporal features (Zhang et al. 2011). Spatial features can be processed using Linear Discriminant Analysis to reduce the dimension of the accumulated feature vector. Since humans recognize the gender of a person from point-light displays of her/his gait and since upper and lower halves of the body provide different contributions (Barclay et al. 1978), spatial processing involves dividing the averaged body silhouette in different body parts and using Support Vector Machine to train the classification weights of all the parts. Temporal features can be processed using Principal Component Analysis (PCA) and Multiple Discriminant Analysis projection to represent individual characteristics in a low-dimensional space and then training a nearest neighbor classifier for identification (Zhang et al. 2011). Deep recurrent neural networks can be trained to detect long-term temporal dependencies for the re-identification of individual gaits (Wu et al. 2016). Notice, however, that the issue of automated surveillance is currently under scrutiny due to the critical ethical considerations raised by the identification of individuals, as well as the potential gender and ethnic biases inherent in some techniques.

## Individual features of movement

### Gait

It has long been known that healthy individuals show considerable differences in walking, even when speed and footwear are controlled (Winter 1988; Simonsen and Alkjær 2012). The individuality principle states that individuals exhibit different motor styles that depend on genetic, developmental and learning processes (Ting et al. 2015). Individual gait features can be identified by means of pattern recognition tools, such as those used in computer gait analysis (see above). A reliable individual characterization requires very large samples of subjects as well as test/retest protocols to verify the persistence of a given feature within individuals at different times, but these criteria are often difficult to satisfy. Two studies examined >100 walking subjects and re-tested a subsample of these subjects a few months (Horst et al. 2017) or years apart (Pataky et al. 2012). These studies succeeded in identifying accurately (classification rate > 99%) the participants based on either plantar pressure (Pataky et al. 2012) or ground reaction force patterns (Horst et al. 2017). Deep artificial neural networks have been used to identify these individual gait patterns reliably (Horst et al. 2019). Hug et al. (2019) were able to label accurately individuals based on the electromyographic (EMG) activity patterns of eight muscles of the lower limbs during gait and pedaling. Avrillon et al. (2018) found that the distribution of activation among the heads of the hamstring muscles is individual-specific.

**Fig. 3:**
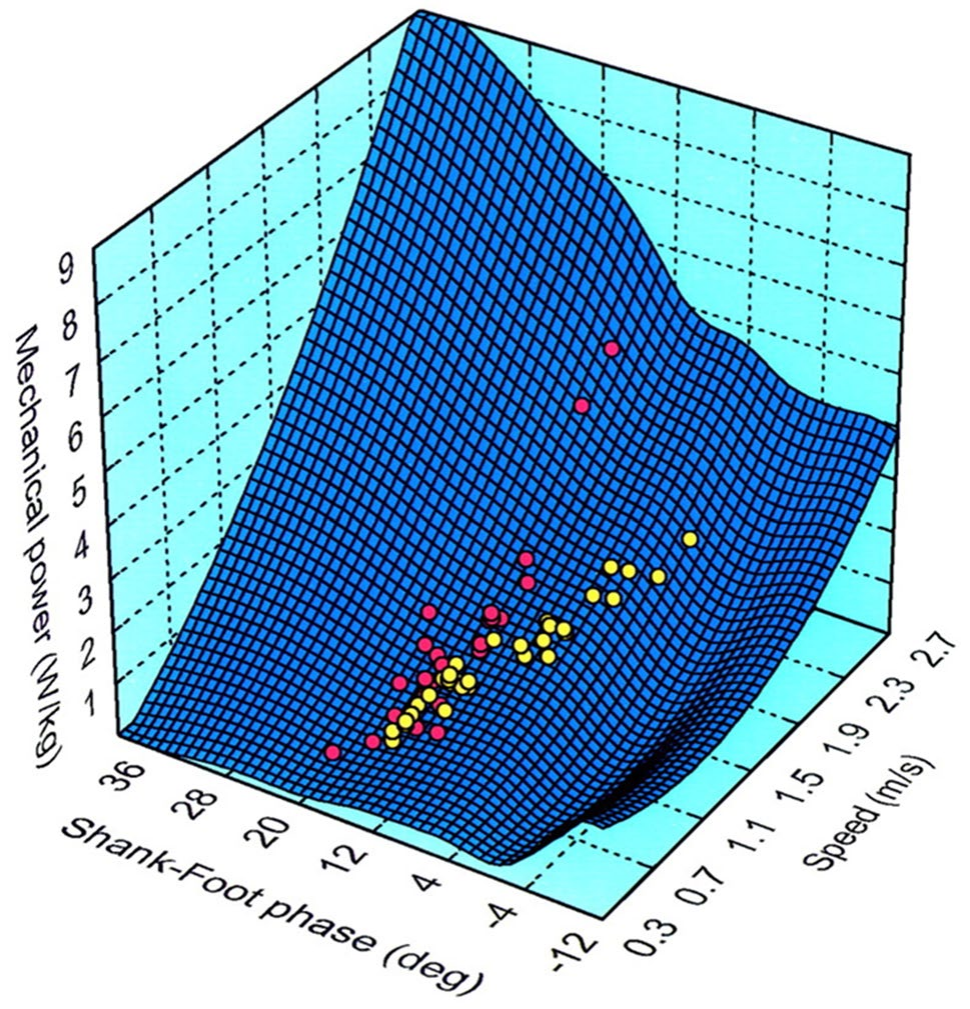
Individual characteristics of walking mechanics. Mass-specific mechanical power is plotted versus walking speed and phase between shank and foot elevation. The curved surface fits the results from 24 different subjects walking at speeds between 0.4 and 2.6 m/s. Individual data from 2 subjects are plotted with different colors: yellow, a trained subject with a pronounced phase shift with increasing speed, red, an untrained subject with a much less pronounced phase shift. Note that the mechanical power output at intermediate and high speeds is correspondingly lower in the former than in the latter. Modified with permission from Lacquaniti et al. (1999)

Another individual feature of walking has been described by considering the intersegmental kinematic coordination. The changes of the elevation angles of the lower limb segments covary along a plane (Borghese et al. 1996; Bianchi et al. 1998). This kinematic law is very robust since it has been confirmed in many animal species in addition to humans, in different laboratories and experimental settings (see Catavitello et al. 2018). However, plane orientation (which depends on intersegmental phase) at any given walking speed has been shown to differ systematically across a sample of 24 healthy human subjects, correlating with the individual expenditure of mechanical energy (Bianchi et al. 1998). In general, the faster we walk, the greater the energy expenditure. The phase coupling between shank and foot provides a compensatory mechanism to reduce the energy fluctuations. However, not all subjects are the same. As shown in Fig. 3, trained subjects (yellow) exhibit a more pronounced phase shift of planar covariation as compared with untrained subjects (red). As a result, trained subjects climb the energy mountain along a less steep, more advantageous path. Interestingly, virtually grafting the kinematics of an energy-saving subject into the body of an energy-hungry subject can save up to 50% of energy in the computed chimera, whereas the opposite (grafting the body of energy-saving subject into the kinematics of energy-hungry subject) does not lead to any saving in the computed chimera (Bianchi et al. 1998). This shows that kinematics is more critical to determine energy expenditure than anthropometric factors such as mass distribution.

A recent study aimed to identify markers defining a person's motor style during posture and locomotion (Mantilla et al. 2020). The hypothesis was that the markers should have two characteristics: first, they should exhibit idiosyncratic features, i.e. they should have the lowest possible intra-individual variability; second, they should be as different as possible between individuals, i.e. they should have the greatest possible inter-individual variability. A person's motor style may affect all of a person's motor activities, but Mantilla’s study was limited to markers that characterized resting postural control and locomotion, behaviors that are regularly scrutinized in the clinic.

**Fig. 4:**
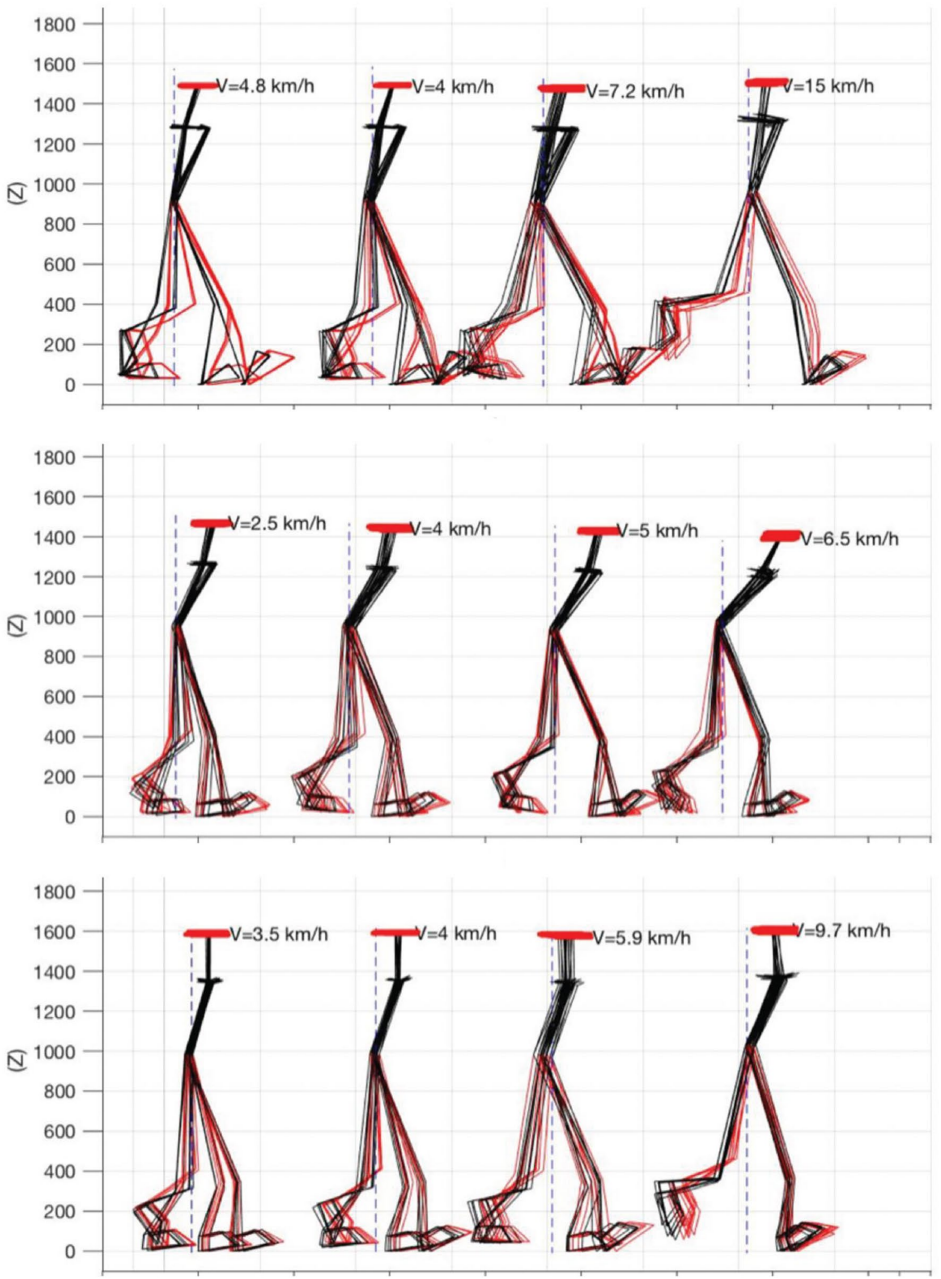
Stick diagrams depicting postural configurations of three subjects (from topo to bottom) in the sagittal view. From left to right: comfortable walk, walk at 4 km/h, race walking and running. Bold red horizontal bars indicate head excursion, while the other lines indicate trunk and leg motions. The configurations have been acquired at the time of heel strike for each foot (red and black overlapped leg configurations). Modified with permission from Mantilla et al. (2020)

Locomotion includes progression towards a goal (navigation), the generation of musculoskeletal patterns to achieve this (dynamic components) while maintaining a stable posture in different environments (static components). Many markers are therefore available to capture the different facets of the style during walking and running. The choice focused on the study of the configuration of the body in the sagittal plane (Fig. 4) and on four dynamic parameters, explored in the transverse (horizontal), sagittal and frontal planes: a measure of the fluidity of the movement expressed by jerk (second time derivative of velocity), a measure of its variability (root-mean-square deviation, RMS), a measure of its regularity estimated by entropy, and a measure of optimization of the trajectory inferred by the relationship between the curvature of movement and its tangential velocity (the two-thirds power law, Lacquaniti et al. 1983). The results confirmed that at rest and during locomotion, motor control in humans can be broken down into two components. A static component is defined by the stable configuration adopted by a given person to position their head, trunk and limbs in relation to gravity. A dynamic component characterizes the relative movements of the head, trunk, arms and limbs. By quantifying and comparing these static and dynamic components, the study was able to identify the set of markers defining motor style during posture and locomotion. They are listed in Table 1. The identification of individual markers of gait has recently been shown to help detecting steps in individuals with severely altered gait due to Multiple Sclerosis (Vienne-Jumeau et al. 2020). A still unresolved issue, however, is whether the individual features of motor style remain stable across the lifespan or change, since several motor control parameters undergo wide changes in one person’s life.

**Table 1:** Summary of features defining motor style during posture and locomotion

	Frontal				Sagittal				Transverse			
	Head	Trunk	Legs	Feet	Head	Trunk	Legs	Feet	Head	Trunk	Legs	Feet
Inclination at rest					+	+						
Inclination for locomotion					+	+						
Jerk locomotion		+	+									
RMS locomotion									+	+		
Entropy locomotion			+	+	+	+			+	+		

Crosses indicate statistically significant inter-individual differences

### Eye movements

Individual motor signatures have also been uncovered for eye movements (e.g., Ettinger et al. 2003; Smyrnis 2008). In a recent study, a 21-dimensional vector of performance metrics of 1058 participants was computed for video-based eye-tracking tasks involving pro-saccades, anti-saccades, and smooth pursuit (Bargary et al. 2017). The biometric parameters were able to identify the oculo-motor signatures of individual participants as shown by re-testing a randomly selected subsample (*n* = 105) of the participants about 20 days after the first session. The importance of biometric parameters of eye movements is also underlined by a study involving eye-tracking of visual targets bouncing back and forth under gravity or artificial reversed gravity (Meso et al. 2020). Grouping participants by high or low traits of schizotypy -assessed by a standard personality questionnaire- showed a negative relationship between schizotypy traits level and both initiation and maintenance of eye-tracking, a result consistent with trait-related impoverished sensory prediction. Divergence of performance between the two groups was especially high for tracking gravity-incongruent targets (Meso et al. 2020).

### Arm movements

Interception of fast targets, such as a tennis serve or a baseball pitch, requires efficient processing of incoming visual information along with prior models of the throw and programming the appropriate response. There are very large differences across individuals in the sensitivity to different types of dynamic visual cues. For instance, Regan and Beverley (1979) found an 80:1 range in the relative sensitivity to retinal dilatation rate and binocular disparity across five tested subjects. Both motion planning and execution are influenced by sensory-motor noise in a highly subject-specific manner (Zago et al. 2009). Thus, systematic differences in several kinematic parameters of interception movements have been reported across naïve subjects reflecting different interception styles (Cesqui et al. 2012; La Scaleia et al. 2015). In one study (Cesqui et al. 2012), participants had to catch on the fly a ball projected by a motorized apparatus with different launch parameters, resulting in different arrival flight times and height conditions. A subset (*n* = 6) of all participants exhibited quite comparable interception performances, and nevertheless, their arm and hand movements differed drastically in several parameters, such as wrist trajectory, wrist velocity profile, timing and spatial distribution of the impact point, upper limb posture, trunk motion, and sub-movement decomposition. Importantly, the individual behaviours were consistent across two experimental sessions carried out at 1-year distance. In a different study (Golenia et al. 2014), the participants learned to pick up a wooden cylinder with different kinds of pliers, a difficult task. The tool grasping profiles of different individuals differed, as did the learning curve during practice.

Fast and efficient visual decoding of throwing styles is especially critical in ball games (e.g. baseball, cricket, etc.). Fast balls afford very little time to process visual information about the trajectory of the approaching ball, given the conspicuous visuomotor delays (Zago et al. 2009). Thus, a 200 km/h tennis serve or a 150 km/h fastball in baseball leave less than 500 ms to the receiver to react, but the brain takes about 250 ms to process ball motion and move accordingly. In fact, sport science has shown that expert players can pick up advance information about the forthcoming ball trajectory and velocity from the observed throwing action of their opponent (Muller et al. 2006; Abernethy et al. 2008; Aglioti et al. 2008), so as to optimize their interception/catching performance (Mann et al. 2010). Indeed, the thrower often tries to limit the involuntary information provided to the receiver by concealing her/his throwing direction.

Maselli et al. (2017) assessed which parameters of whole-body kinematics of the thrower best correlate with the direction of a thrown ball. To this end, they recorded the throwing actions of 20 non-experts asked to hit one of four targets at 6 m distance. By using dimensionality reduction and machine learning techniques, they found that the throwing arm provides accurate information about the outgoing ball trajectory, but only in the very last phase of the throwing action, at 100–200 ms before ball release. At earlier times prior to ball release, the trunk and the upper and lower limbs contralateral to the throwing arm provide informative cues. This study also detected differences in throwing styles across the sample of throwers, with corresponding inter-individual differences in the spatio-temporal structure of the thrower’s predictability. For most participants, fairly accurate predictions of where in space the ball will land could be reached as early as 400–500 ms before ball release from the hand.

**Fig. 5:**
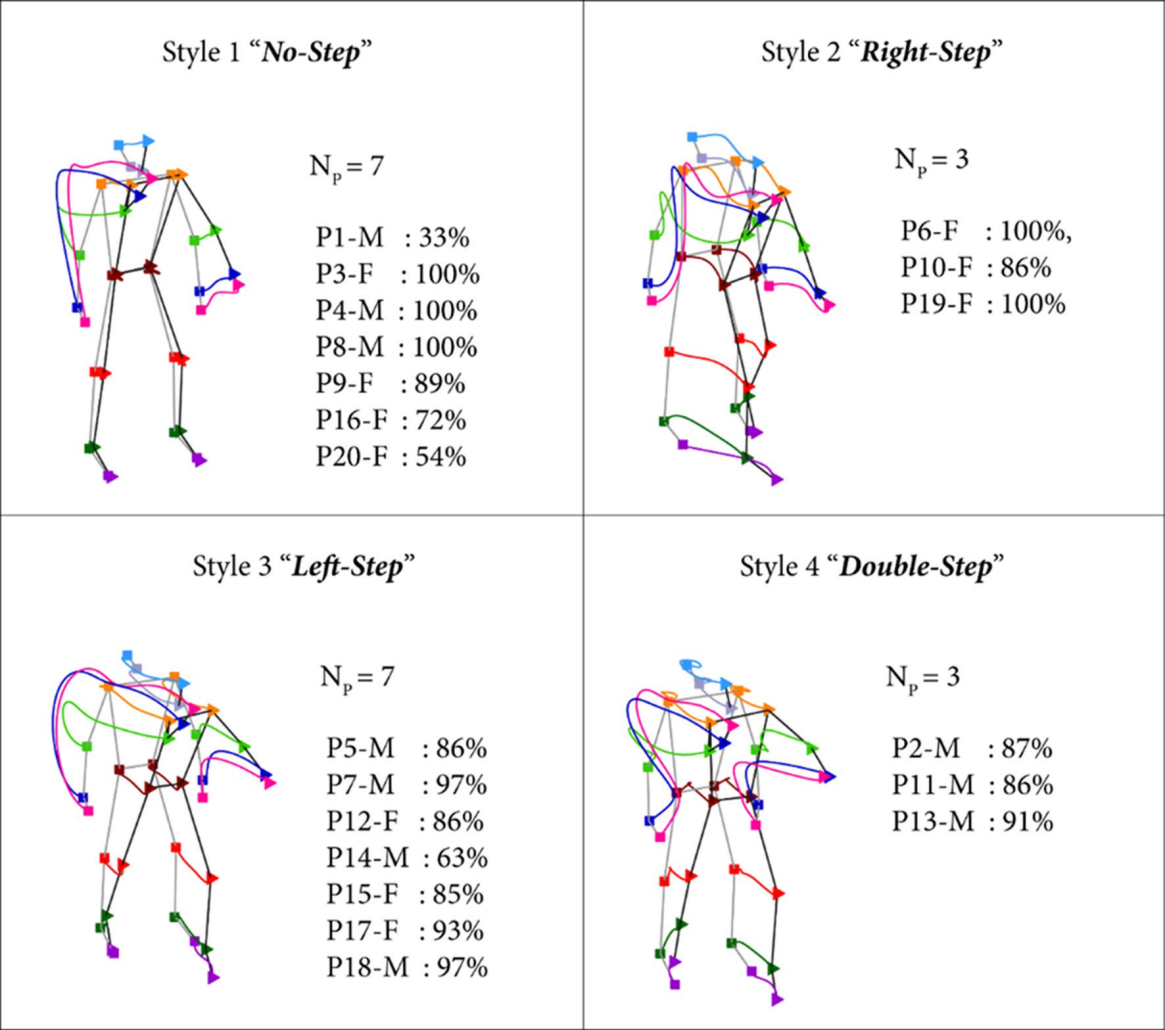
The four typical throwing styles emerging from cluster analysis. Each panel shows the mean throwing trajectories averaged across all trials assigned to the corresponding cluster, independently of the individual thrower. Different colors correspond to different joint markers. Throwing styles can be adopted by different throwers. Each panel further reports the number of participants for whom the highest fraction of throws is assigned to the corresponding style (*N*_P_), the participant identity (P1–P20) and gender (M, F), and the fraction of throws that is assigned to that specific throwing style represented in the panel. Modified with permission from Maselli et al. (2019)

The individual throwing strategies were specifically investigated by Maselli et al. (2019). They found that the identity and gender of the thrower could be reliably inferred from the kinematics of a single throw. In particular, cluster analysis identified four main classes of throwing strategies (motor styles), which were very consistent within individuals. The four styles consisted in no-step, right-step, left-step, and double-step prior to the throwing arm gesture, these stepping movements taking place at various times prior to the throw (Fig. 5). Interestingly, these styles were reminiscent of the throwing modes exhibited by children during the main stages of proficiency acquisition during motor development (Wild 1938; Roberton et al. 1979). Thus, the results support the idea that inter-individual and gender differences in skilled behaviour, such as throwing, are related to skill acquisition interrupted at different stages of the typical developmental trajectory of the specific motor behaviour. However, these results are still preliminary and we still do not know whether the different styles are correlated with a different performance success.

Hilt et al. (2016) reported large inter-subject variability as compared with intra-subject variability in whole-body reaching movements towards a surface. They found that individual motor styles spanned a continuum between the two extreme patterns of ankle and knee strategies. Hilt and colleagues were able to account for the idiosyncratic behaviours by means of individual combinations of different optimality criteria, involving mechanical energy expenditure, joint smoothness and minimization of the amount of torques. Słowiński et al. (2016) identified individual motor signatures in freehand movements by clustering the distances between the velocity profiles of different participants. They further showed that coordination between two individuals performing a joint-action task was higher if their motions shared similar dynamic features. However, there are conditions under which participants cannot avoid (unintentionally) coordinating with someone else, and to do so they must give up their original movement pattern (Issartel et al. 2007).

A recent study (Sternad 2018) points out that variability in motor performance represents both a nuisance and an advantage. On the nuisance side, aging by causing changes in both musculoskeletal, vestibular, and visual receptors can increase variability in gait, leading to instability and falls (Herssens et al. 2018; Kikkert et al. 2016; Ayoubi et al. 2015), particularly in frail elderly people (Schwenk et al. 2014; Dasenbrock et al. 2016; Mortaza et al. 2014; Herssens et al. 2018). The variability of gait also increases during various pathologies (Figueiredo et al. 2018) such as cerebellar and vestibular ataxia (Schniepp et al. 2017; Buckley et al. 2018) and neurological disorders with motor deficits (Ivanenko et al. 2013; Moon et al. 2016).

However, variability in motor performance can also be an advantage and depends in particular on the structure of the motor task. Motor components that contribute directly to the task often show little variability, while components that do not contribute tend to be more variable, thus reducing the adoption of different speeds leading to changes in the variability of the components of the stride, changes that have an adaptive value (Dingwell and Cusumano 2015; Dingwell et al. 2017, Möhler et al. 2020). In addition, as a comparison between experienced and novice runners shows, variability during the stride could reduce the risk of injury (Mo and Chow 2018; Hamill et al. 2012). Variability may also specifically contribute to the acquisition of novel perceptual-motor behaviours, such as when walking on a split-belt treadmill (Van de Putte et al. 2006, Altman et al. 2012) or on a narrow beam (Sawers et al. 2015), as well as during pathologies that affect the locomotor system (Mawase et al. 2016). Finally, the variability of several parameters during walking are good indicators useful for the study of sensorimotor development (Kraan et al. 2017).

The studies reviewed above show that humans exhibit various styles of perception and motor behavior, which are based on inter-individual variations in the way they treat sensorimotor transformations. In this context, the characterization and monitoring of the perceptual-motor style are important for several reasons. First, predictions are important for monitoring many aspects of human behavior when individuals interact with each other. This requires prior knowledge of relevant sources of information to make reliable predictions about the behavior of others, predictions that vary according to the perceptual-motor style of the person with whom one interacts (Maselli et al. 2019).

Second, differences in perceptual-motor style, since they are idiosyncratic, can be more or less efficient, raising the question of when they need to be adjusted to maintain optimal control (see Moore 2016 for a review of the economy of walking and running). Third, changes in a person's perceptual-motor style could reveal the beginning of a pathological process and help to track their recovery (König et al. 2016).

## Style may depend on age

Manipulation of sensory information induces postural changes even in infants, and this ability to use sensory information increases up to ten years of age (Delorme et al. 1989), Higgins et al. 1996; Barela et al. 1999; Bertenthal et al. 2000; Barela et al. 2000, 2003; Schmuckler 1996; Godoi and Barela 2008) and even beyond (Godoi and Barela 2008, Peterson et al. 2006; Sparto et al. 2006; Zernicke et al. 1982). Infants and children, however, have more difficulty in resolving sensory conflict situations correctly and may even fall (Lee and Aronson 1974; Forssberg and Nashner 1982; Stoffregen et al. 1987). In particular, children under the age of 7 years have difficulty integrating sensory information correctly and favor visual information (Lee and Aronson 1974; Forssberg and Nashner 1982; Rival et al 2005; Shumway-Cook and Woollacott 1985; Wolff et al. 1998; Woollacott et al. 1987) but this dominance is open to debate (Barela et al. 2003, Godoi and Barela 2008; Metcalfe et al. 2005; Peterson et al. 2006; Bair et al. 2007). Children, to a certain extent, would be able to weight sensory information to control their posture (Barela et al 1999, 2000,2003; Schmuckler 1997; Polastri and Barela 2013). However, these adaptations have their limits below 12 years of age (Lee and Aronson 1974; Butterworth and Hicks 1977; Forssberg and Nashner 1982; Wann et al. 1998; Rinaldi et al. 2009; Polastri and Barela 2013). Using portable devices, a few studies explored the motor signatures of developmental disorders, such as autism (Jansiewicz et al. 2006; Anzulewicz et al. 2016).

As remarked above, elderly and young individuals learn a task by taking advantage of different aspects of motor variability (Cheung et al. 2020). As we age, the sharpness of our senses diminishes, and this can affect our lifestyle. To compensate for this deterioration, the brain reweighs the sources of sensory information according to their signal-to-noise ratio. Numerous studies suggest that the degeneration of the vestibular system (Rosenhall and Rubin 1975) and the accompanying decrease in the signal-to-noise ratio of vestibular information may explain the preponderance of visual information during aging (Anson and Jeka 2016; Jeka et al 2006; Alberts et al. 2019) and in accompanying pathologies (Bronstein et al. 1996; Bronstein 1999; Guerraz et al. 2001; Lopez et al. 2007; Grabherr et al. 2011). This process of reweighting sensory information in favor of visual inputs would include the estimate of the vertical direction (Curthoys 2000; Peterka 2002; Peterka and Loughlin 2004). This primacy of visual information is also explained by the deterioration of proprioceptive inputs with age (Deveze et al. 2014; Iwasaki and Yamasoba 2014; Clemens et al. 2011; Alberts et al. 2016). The biological mechanisms at play are multiple. Recently, Karmali et al. (2017, 2018) explained changes in gaze stabilization strategies with age (Dimitri et al. 2001) as an adaptation to the gradual disappearance of hair cells from semicircular canals. Similarly, adaptations of postural control would be initiated by the loss of utricular and saccular hair cells. A priori, cell loss in the five vestibular sensors would progress at the same rate (Gleeson and Felix 1987; Matheson et al. 1999).

## Style and learning

When a new task in an unstable environment is learned, the CNS must find a motor strategy that reduces the risk of errors, while remaining energy efficient (Ter Horst et al. 2015). This learning is accompanied by a reweighting in the processing of sensory input. For example, in the case of a unipedal learning task on an unstable surface, van Dieën et al. (2015) showed that the initial presence of postural oscillations, with training, became associated first with an increase in the weighting of visual information, and then with a decrease in the weighting of proprioceptive information. As another example, tightrope walkers make extensive use of rapid head and trunk movements to maintain balance and a significant weighting of proprioception of neck and lumbosacral regions (Honegger et al. 2013). The reader interested in the reweighting of sensory information in the athlete can also refer to several studies on the subject (Kioumourtzoglou et al. 1998; Paull and Glencross 1997; Bringoux et al. 2000, Vuillerme et al. 2001; Hamill et al. 2012; Busquets et al. 2018; Mo and Chow 2018). The conclusion of Thalassinos et al. (2018) is interesting to conclude this point: each sport would favor a particular weighting in the use of sensory information. Tightrope walkers and dancers favor proprioceptive afferences, footballers favor visual afferences etc. Notice that studies in the field of sports science have described differences between professionals and novices, differences between different kinds of sport, and also differences among experts of the same sport (e.g., Nasu et al. 2014). Finally, Smyth et al. (2019) have shown that the reduction in cortisol reactivity to psychosocial stress in healthy women is linked to a greater visual dependence in postural control, which opens up a vast field of study that remains to be explored on the links between affect, stress and sensory weighting.

## Mechanistic bases

The origin of perceptual-motor styles is still as mysterious as the origin of painting styles of Monet or Renoir. In line of principle, inter-individual differences in sensorimotor neural circuitries and their coupling with peripheral mechanics may be shaped by genetics, development, motor exploration, experience, training and/or pathology. Some progress has been made toward identifying elements that may contribute to creating inter-individual differences in sensorimotor performance, such as a different conformity to optimal training (Bianchi et al 1998), different adherence to distinct developmental stages (Maselli et al. 2019), learning strategy (Pacheco and Newell 2018) plus memory during task practice (Ganesh et al. 2010; Loeb 2012), and gene-mediated factors (Williams and Gross 1980). In particular, significant genetic effects on both performance levels and rates of improvement have been suggested by comparing monozygotic with dizygotic twins in a variety of tasks, including manual tracking, tapping speed, reaching, and balance, with heritability values ranging between about 20 and 50% as a function of the task (Williams and Gross 1980; Fox et al. 1996; Missitzi et al. 2013; Zempo et al. 2017; Christova et al. 2020). However, the relative importance of genetic variation in skill development remains controversial (Yarrow et al. 2009).

Irrespective of the extent to which individual styles depend on genetic factors, one may ask the question of the developmental stage at which sensorimotor patterns become unique (Gandevia et al. 2019). Healthy human newborns exhibit considerable variability in their spontaneous movements, such as leg kicking or arm flailing (Sylos-Labini et al. 2020). However, we still do not know how distinct these motor patterns are, whether they exhibit individual features, how they develop over time and become truly idiosyncratic of each person.

The number of studies specifically addressing the mechanistic underpinnings of style is still limited. One approach consists of investigating the individual neural strategies involved in the control of a motor task. In a recent study (Avrillon et al. 2020), high-density surface electromyography recordings were decomposed into motor unit action potentials for a task involving submaximal isometric knee extensions. The results showed that the neural strategies to control two knee extensor muscles (vastus lateralis and vastus medialis) varied widely across individuals, the individual strategies being consistent across sessions interspaced by 20 months. Specifically, the distribution of the strength of neural drive between the vastus lateralis and vastus medialis, as well as the proportion of neural drive shared within and between these muscles varied across participants. The coordination of vastus lateralis and vastus medialis is important for the regulation of the internal stress forces of the knee joint (Alessandro et al. 2020). Accordingly, a large common drive between these muscles observed in the majority of the participants of the study by Avrillon et al. (2020) might represent an efficient strategy to prevent knee injury. By contrast, the lower common drive observed in a minority of participants might be associated with a higher risk of developing knee-related injuries.

The neural basis of the individual differences in locomotion (e.g., Bianchi et al. 1998; Hug et al. 2019; Mantilla et al. 2020) is still unknown. Individual differences may arise from, among other factors, the almost unlimited potential combinations of neural activity due to variable, dynamic reconfiguration of the circuits (Marder 2011) and their redundant organization (Hultborn 2001). Thus, a recent study showed a striking redundancy in the spinal locomotor networks of a mouse model (Pham et al. 2020). Using differential labelling of spinal interneurons, the study showed that between two 30-min bouts of stepping, each consisting of thousands of steps, only ~ 20% of the neurons activated from the first bout of stepping were also activated by the second bout. This finding suggests that variability of neural networks organization may enable the selection of many different combinations of neurons when generating each step cycle.

Other interesting approaches to investigate putative neural substrates of individual perceptual-motor styles involve the description of individual patterns of brain activity in humans. By correlating kinematics and fMRI responses, it has been shown that kinematic variability and parietal and prefrontal cortical variability are stable individual traits, consistent across movements to different targets when performed by either the right or left arm (Haar et al. 2017). The same study also showed that subjects with larger neural variability in the inferior parietal lobule have larger movement–extent variability. Another fMRI study scanned a reinforcement learning task in which participants stopped a rotating clock hand to win points (Badre and Frank 2012). The results showed that the pattern of activity in rostrolateral prefrontal cortex distinguished individuals who rely on relative uncertainty for their exploratory decisions versus those who do not. Another recent study (Xue et al. 2021) used functional connectivity MRI to examine the cerebellum of two intensively-sampled individuals (each scanned 31 times) and found idiosyncratic spatial details between these subjects.

Hilt et al. (2020) addressed the issue of how individual motor styles are dealt with during action observation. They asked participants to first perform and then observe a whole-body reaching action that could be performed according to several different styles, generally spread within a continuum between two extreme strategies (see above, Hilt et al. 2016). Then, they measured the corticospinal excitability of the participants by applying transcranial magnetic stimulation on the motor cortex while the participants observed an actor achieving the same goal by using the two extreme strategies of action. They found that the individual corticospinal excitability was an inverse function of the distance between the observer’s style and the actor’s style, in other words, the corticospinal excitability was greater the closer were the observer’s style and the actor’s style.

## Perspectives

As this review has attempted to broadly summarize, the perceptual-motor style may vary from one individual to another, from one task to another, from one pathology to another, as sensorimotor transformations show considerable adaptability and plasticity. While the behavioral evidence for individual styles is already quite significant, much work remains to be done to understand the neural and mechanical substrates of the inter-individual differences in sensorimotor performance.

It should also be stressed that the fact that the perceptual-motor style may change during intensive physical activity or during the course of a disease does not in any way guarantee that it is for the benefit of the athlete or the patient. Again, functional or post-injury plasticity, while well established, has not been proven to be effective or harmful when it occurs spontaneously. On the other hand, numerous studies also show that the perceptual-motor style can evolve with proactive learning. Whether we talk about training in sports or re-education in patients, the issues are similar.

In this context, we plead for training, learning and rehabilitation to be the subject of longitudinal studies so that they can be optimized for the benefit of athletes and patients. The identification of perceptuo-motor styles via the quantification of reliable markers of individual behavior would help considerably to develop personalized treatments. This goal seemed almost unattainable until recently, because it involves the detailed quantification of a wide range of critical performance parameters in normal and pathological human behavior in realistic settings. Recent progress with intensive computational methods now makes the goal within our reach.

Finally, it must be stressed that the study of sensorimotor transformations and the perceptual-motor style has important implications for rehabilitation practice. Rehabilitation, for purely economic reasons, is still largely under-dosed. With the ageing of the population and the problem of maintaining autonomy, this policy is no longer tenable (Vidal et al. 2020). Personalized approaches along with precision diagnostics will pave the way to many improved treatments.
